# Integration of the Pirogov interactive anatomy table into anatomy teaching: A comparative study with cadaveric dissection

**DOI:** 10.1371/journal.pone.0341597

**Published:** 2026-01-28

**Authors:** Nguyen Thien Duc, Nguyen An Ninh, Nguyen Phi Trinh, Le Quang Tuyen, Nguyen Van Hung, Dinh Hoang Khanh, Nguyen Van Luat, Nguyen Huu Phuc Dai, Tran Duc Huy, Chu Duc Hoa, Tran Vuong The Vinh

**Affiliations:** 1 Department of Anatomy and Embryology, University of Health Sciences, National University at Ho Chi Minh City, Ho Chi Minh City, Vietnam; 2 Department of Orthopedics, Thong Nhat Hospital, Ho Chi Minh City, Vietnam; 3 Department of Anatomy, Pham Ngoc Thach University of Medicine, Ho Chi Minh City, Vietnam; 4 Department of Surgery, Hai Phong University of Medicine and Pharmacy, Hai Phong, Vietnam; Universidad Complutense de Madrid, SPAIN

## Abstract

**Purposes:**

Anatomy is fundamental in medical education, yet cadaveric dissection faces challenges including limited specimens, high costs, and chemical hazards. Interactive anatomy tables such as the Pirogov system offer innovative alternatives, but evidence from Southeast Asia is limited.

**Methods:**

In a prospective cohort, 188 medical students (139 in Y1 and 49 in Y2) were randomly assigned to the Pirogov table group (Group A, n = 99) or the cadaveric dissection group (Group B, n = 89). Knowledge acquisition was measured using a validated 20-item multiple-choice test before and after the intervention. Student perceptions were evaluated with a 10-item Likert-scale questionnaire covering four domains: knowledge and understanding, spatial visualization and relationships, learning experience and engagement, and effectiveness and practical value. Data were analyzed using paired and independent t-tests and Welch’s t-test.

**Results:**

Both groups showed significant knowledge gains (Group A: 4.3 ± 1.65 to 5.2 ± 1.75, p < 0.001; Group B: 4.2 ± 1.92 to 5.1 ± 1.64, p < 0.001), with no difference between them (p = 0.656). Likert ratings were consistently high across domains, with means from 4.43 to 4.48. Y1 students reported higher ratings than Y2 in learning experience (p = 0.023).

**Conclusion:**

The Pirogov table and cadaveric dissection were associated with similar short-term improvements in anatomy knowledge. Students valued the Pirogov table for visualization and engagement. These findings support integrating digital tools with cadaveric dissection to enhance anatomy education, particularly in resource-limited contexts.

## Introduction

Anatomy is a cornerstone of medical education, providing essential knowledge for clinical reasoning and surgical training. For decades, cadaveric dissection has been regarded as the “gold standard” in anatomy teaching because it offers direct, three-dimensional and tactile experiences of the human body. However, this traditional approach faces growing challenges: limited body donation, high costs of preservation, health hazards from chemical exposure, and a global reduction in teaching hours allocated to anatomy [1–4].

Advances in educational technology have introduced interactive anatomy tables such as Anatomage, Sectra, and the Pirogov system. These platforms allow visualization of detailed three-dimensional structures, virtual dissection across multiple planes, and integration of teaching modules. Studies from high-income countries have reported positive student perceptions, particularly regarding spatial understanding and engagement [5–7]. Yet, evidence on whether such tools can match cadaveric dissection in knowledge acquisition remains inconclusive [8].

Importantly, most available data come from Western settings, while evidence from low- and middle-income countries is scarce [8]. In contexts like Vietnam, where access to cadavers is restricted and resources are limited, the educational value of digital anatomy platforms has not been systematically evaluated. To our knowledge, no controlled study has compared the Pirogov interactive table with cadaveric dissection in this region.

The present study aimed to address this gap by comparing knowledge acquisition between students taught with cadaveric dissection and those taught with the Pirogov’s interactive anatomy table (Pirogov, Samara, Russia) and assessing student perceptions of the Pirogov table. By providing context-specific evidence from Vietnam, the study can contribute new insights to the global debate on how best to integrate digital tools into anatomy curricula.

## Materials and methods

### Study design and setting

This was a prospective study, conducted at the Faculty of Medicine, Vietnam National University Ho Chi Minh City (April 2024–April 2025). The research protocol was reviewed and approved by the Institutional Review Board of the Faculty of Medicine, Vietnam National University Ho Chi Minh City (Approval No. 05/QD-IRB-VN01.017, dated March 29, 2024). Participation was voluntary, and all students provided informed consent.

### Participants

First- and second-year medical students (Y1 and Y2) with no prior formal anatomy training were eligible. Inclusion required completion of the baseline pre-test and provision of informed consent after receiving detailed study information. Students were excluded if they missed the post-test, failed to submit the perception survey, or were absent from scheduled sessions. Randomization generated in Microsoft Excel using a fixed random seed and permuted blocks of four. Allocation was concealed in sequentially numbered, opaque, sealed envelopes by an independent administrator. Test scoring and data analysis were performed by assessors blinded to group assignment. All 188 students completed the pre-test, post-test, and survey; no imputation was required.

### Sample size calculation

The sample size was calculated to detect a mean difference of 0.8 points with α = 0.05 and β = 0.20 (80% power) [9]. A minimum of 89 participants per group was required. In total, 188 students were enrolled and assigned into two groups:

- Group A (n = 99): Pirogov table
- Group B (n = 89): Cadaveric dissection

### Study procedure

**Fig 1:**
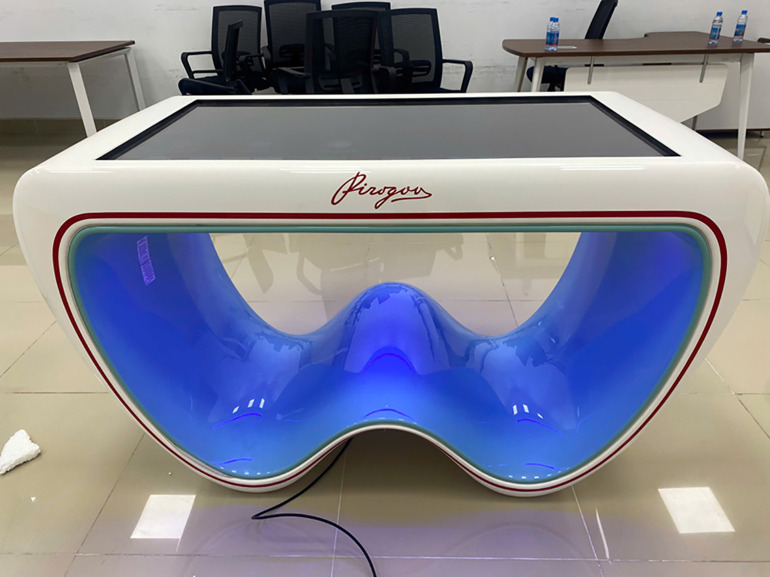
The Pirogov interactive anatomy table.

**Fig 2:**
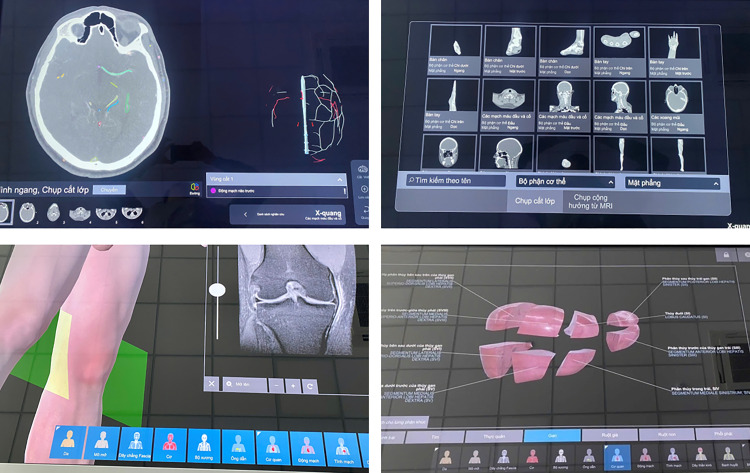
Representative views from the Pirogov interactive anatomy table showing anatomical visualization and virtual dissection functions.

Eligible students were enrolled and completed a validated 20-item multiple-choice pre-test designed by the anatomy faculty (S1 File). Both groups attended two teaching sessions on the musculoskeletal system (1.5 h each). Regarding practice sessions, Group A received instruction using the Pirogov interactive anatomy table (Figs 1 and 2), whereas Group B participated in cadaveric dissection. Teaching duration, content, and instructors were standardized across groups to ensure comparability. Specifically, both groups were taught by the same pair of anatomy instructors, each with over 5 years of teaching experience and formal training in cadaveric dissection and digital anatomy instruction. A standardized lesson plan, including scripted prompts, identical teaching slides, learning objectives, and demonstration sequences, was used for both modalities. Instructor enthusiasm and delivery style were controlled by adhering to a predefined instructional script that minimized variation in tone, pacing, and interaction level. Students then completed a post-test of identical format.

The two teaching sessions were held simultaneously in separate, supervised rooms, preventing students from observing the other modality. Students were explicitly instructed not to discuss teaching content with peers until all assessments were completed. Faculty proctors monitored students before and after sessions to reduce opportunities for cross-sharing. The post-test was administered immediately after the session and before the cross-exposure phase, ensuring no contamination of primary outcome measures. Following the post-test, the groups exchanged practice sessions and engaged in three hours of self-study. Finally, all participants completed a 10-item Likert-scale questionnaire (Table 1) evaluating perceptions of the teaching method.

**Table 1:** Thematic grouping of the 10-item Likert questionnaire.

Group	Description	Related Questions
Knowledge and Understanding	Assesses how cadaveric dissection and the Pirogov table improve students’ comprehension of upper and lower limb anatomy, either independently or in combination.	Q1. Using the Pirogov anatomy table helps me better understand the anatomy of the upper and lower limbs. Q2. Cadaveric dissection helps me better understand the anatomy of the upper and lower limbs. Q3. I gained a deeper understanding of the anatomy of the upper and lower limbs by using the Pirogov anatomy table in combination with cadaveric dissection.
Spatial Visualization and Relationships	Evaluates the ability of the Pirogov table to support recognition of anatomical details in 3D, relative sizes of structures, and the relationships among body parts.	Q4. The Pirogov anatomy table helps me recognize differences in anatomical details of the upper and lower limbs in 3D images. Q5. The Pirogov anatomy table helps me better visualize the relative sizes of anatomical structures in the upper and lower limbs. Q6. The Pirogov anatomy table helps me understand the relationships among different parts of the body.
Learning Experience and Engagement	Captures students’ enjoyment, satisfaction, and motivation when using the Pirogov table during the learning process.	Q7. I enjoyed the entire process of using the Pirogov anatomy table. Q8. Using the Pirogov anatomy table enhanced my learning experience and increased my interest in anatomy.
Effectiveness and Practical Value	Measures perceptions of time efficiency compared with cadaveric dissection and the potential for integrating the Pirogov table into routine anatomy teaching.	Q9. Virtual dissection on the Pirogov anatomy table takes less time compared with cadaveric dissection. Q10. The Pirogov anatomy table should be integrated into regular anatomy teaching.

### Outcome measures

Knowledge acquisition: Assessed using a standardized multiple-choice questionnaire (20 items covering musculoskeletal anatomy). Tests were administered before (pre-test) and after (post-test) the intervention. The questionnaire was designed and validated by anatomy faculty, with good internal consistency (Cronbach’s alpha = 0.82). The 20-item test was analyzed for difficulty index (mean = 0.56) and discrimination index (mean = 0.32), both within acceptable ranges for educational assessments. The pre-test and post-test used identical questions, a common method in short-term knowledge-gain studies. A 20-item MCQ assessment is adequate to detect group-level differences, referencing similar sample sizes and test lengths used in anatomy education trials.

Student perceptions: Evaluated using a 10-item survey on a five-point Likert scale (1 = strongly disagree, 5 = strongly agree), adapted from previously published international studies and modified to the local context [9–12].

### Data statistics

The data was entered and analyzed using the IBM SPSS version 25 (IBM Corp., Armonk, N.Y.). The anonymized raw dataset used for all analyses is provided (S2 File). Descriptive statistics were reported as mean ± standard deviation. Within-group changes in pre-test and post-test scores were examined using paired t-tests, while between-group comparisons of post-test scores were evaluated using independent t-tests.

Subgroup analyses comparing Y1 and Y2 participants were treated as exploratory, as the study was not originally designed to test cohort-level differences and the groups differed substantially in size. To account for unequal sample sizes and potential heterogeneity of variance, Welch’s t-test was used for all subgroup comparisons. These analyses were intended to contextualize perception ratings rather than draw inferential conclusions regarding cohort-level differences.

Baseline comparability between randomized groups was assessed through independent t-tests (continuous variables) and chi-square tests (categorical variables). No statistically significant differences were observed in age, gender distribution, or pre-test scores, suggesting acceptable balance at the start of the study. However, the analysis did not incorporate multivariable regression modeling to control for potential confounders such as year of study, gender, prior informal anatomy exposure, or baseline academic ability. This decision was based on sample size considerations and the risk of model overfitting.

All statistical tests were two-tailed, and a p-value < 0.05 was considered statistically significant. Given the exploratory nature of several analyses, no correction for multiple comparisons was applied, and findings should be interpreted with caution.

## Results

### Participant characteristics

A total of 391 students registered; 188 met eligibility and completed all procedures (Y1 = 139; Y2 = 49). The overall mean age was 18.3 ± 0.9 years old (range, 17–25 years old). The gender distribution of the total sample was 112 females (59.6%) and 76 males (40.4%).

The demographic characteristics of the two study groups were comparable. The mean age was 18.4 ± 1.0 years in Group A and 18.2 ± 0.7 years in Group B (p = 0.114). Gender distribution was also similar, with 37 males and 62 females in Group A compared with 39 males and 50 females in Group B (p = 0.453). These findings indicated that the two groups were well balanced in terms of age and gender at baseline.

### Knowledge acquisition and correlation between pre-test and post-test scores

**Fig 3:**
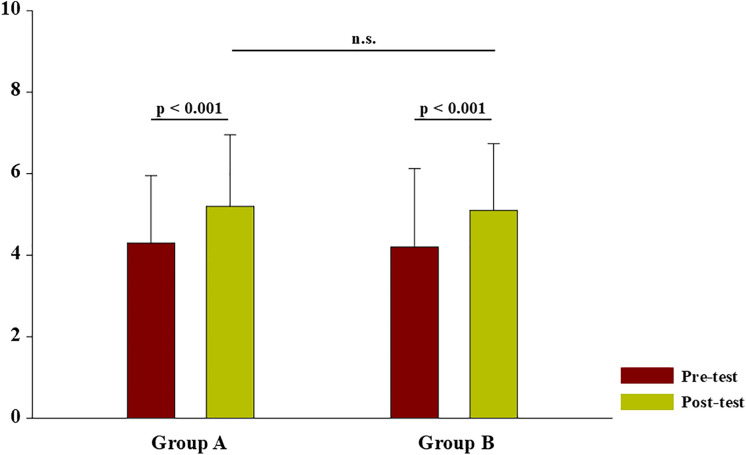
Pre-test and post-test knowledge scores by teaching modality (Pirogov table vs cadaveric dissection). The graphs showed mean pre-test and post-test scores for Group A (Pirogov interactive anatomy table; n = 99) and Group B (cadaveric dissection; n = 89). Both groups demonstrated significant improvements from pre-test to post-test (paired t-test, p < 0.001 for each group). Post-test scores were not significantly different between groups (independent t-test, p = 0.656; n.s.). Error bars indicate standard deviation (SD).

Both groups demonstrated significant improvements in knowledge acquisition from 4.3 ± 1.65 to 5.2 ± 1.75 (95% CI, –1.195 to –0.438) in Group A and from 4.2 ± 1.92 to 5.1 ± 1.64 (–1.217 to –0.331) in Group B (both p < 0.001). However, when comparing post-test scores between the two groups, no statistically significant difference was observed (p = 0.656), indicating that the effectiveness of the Pirogov table and cadaveric dissection was comparable (Fig 3).

A moderate positive correlation was observed between pre-test and post-test scores in both groups (Group A: r = 0.376; Group B: r = 0.312; both p < 0.05), suggesting consistent improvement across different baseline levels of student performance.

### Student perceptions

Analysis of Likert-scale responses revealed consistently high ratings across all four domains, with mean scores ranging from 4.43 ± 0.80 to 4.48 ± 0.82 on a five-point scale (Table 2). The highest ratings were observed in Spatial Visualization and Relationships (4.48 ± 0.77) and Learning Experience and Engagement (4.48 ± 0.82), indicating that students particularly valued the Pirogov table for enhancing three-dimensional visualization and enriching the learning process.

**Table 2:** Overall Likert-scale domain scores of participants (n = 188).

Variable	Mean ± SD
Knowledge and Understanding	4.45 ± 0.75
Spatial Visualization and Relationships	4.48 ± 0.77
Learning Experience and Engagement	4.48 ± 0.82
Effectiveness and Practical Value	4.43 ± 0.80

SD, standard deviation.

**Fig 4:**
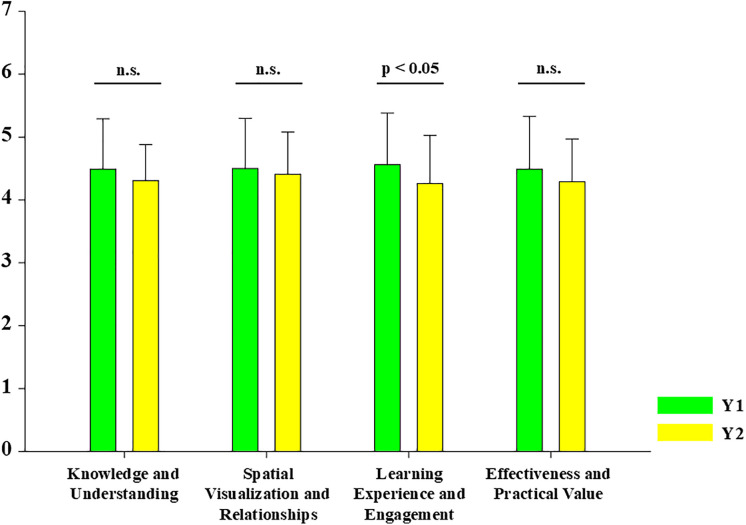
Likert-scale domain scores by cohort (Year 1 vs Year 2 medical students). The graphs presented mean domain scores (± SD) from the 10-item Likert questionnaire comparing Year 1 (Y1; n = 139) and Year 2 (Y2; n = 49) students across four domains: Knowledge and Understanding; Spatial Visualization and Relationships; Learning Experience and Engagement; Effectiveness and Practical Value. Between-cohort comparisons were performed using Welch’s t-test due to unequal sample sizes. A statistically significant difference was observed only for Learning Experience and Engagement (p = 0.023), while other domains were not significant (Knowledge and Understanding p = 0.095; Spatial Visualization and Relationships p = 0.428; Effectiveness and Practical Value p = 0.099; n.s.). Error bars indicate standard deviation (SD).

By cohort, Y1 students reported higher ratings than Y2 in all domains: Knowledge and Understanding (4.49 ± 0.80, 4.31 ± 0.57, p = 0.095), Spatial Visualization and Relationships (4.50 ± 0.80, 4.41 ± 0.67, p = 0.428), Learning Experience and Engagement (4.56 ± 0.82, 4.26 ± 0.77, p = 0.023 < 0.05), and Effectiveness and Practical Value (4.49 ± 0.84, 4.29 ± 0.68, p = 0.099) (Fig 4).

## Discussion

This study evaluated the comparative effectiveness of cadaveric dissection and the Pirogov interactive anatomy table in undergraduate medical education. Both modalities produced significant improvements in anatomical knowledge, with comparable post-test outcomes and no statistically significant difference between groups. These findings suggest that digital anatomy platforms can achieve similar short-term knowledge gains to cadaveric dissection under standardized instructional conditions, while also offering distinct advantages in visualization and student engagement.

Our results are consistent with prior studies demonstrating that interactive anatomy tables yield learning outcomes similar to cadaveric dissection. Anand et al. [9] reported no significant differences in neuroanatomy knowledge between students taught using the Anatomage table and those using traditional cadavers, though students appreciated the digital platform’s interactive features. Kausar et al. [13] similarly found that Sectra table instruction did not surpass cadaver-based learning in test scores, but improved attention and engagement. By situating our findings in the Vietnamese context, this study expands the evidence base to a low- and middle-income setting where cadaver resources are scarce and cost-effectiveness is a crucial concern.

Students’ perceptions of the Pirogov table were overwhelmingly positive, echoing international literature. Brown et al. [14] highlighted that medical students valued interactive 3D anatomy platforms as highly engaging tools for visual learning. Tenaw et al. [15] also documented strong student acceptance of digital anatomy teaching in Ethiopia, underscoring its motivational impact. In the study, the highest ratings were observed in domains related to spatial visualization and learning experience, suggesting that interactive tools may particularly enhance students’ ability to conceptualize complex anatomical structures and foster enthusiasm for learning. Notably, Y1 students reported significantly higher scores in the domain of learning experience compared with Y2. This difference may reflect the novelty effect and greater initial motivation among first-year students, while more advanced students with prior exposure to cadaveric methods may have evaluated the tool with a more critical perspective. However, this difference should be interpreted with caution given the unequal cohort sizes, despite the use of Welch’s test.

From an educational theory perspective, these findings can be interpreted through cognitive load theory and multimedia learning principles. Interactive 3D visualization may reduce extraneous cognitive load by clarifying spatial relationships and supporting spatial cognition (e.g., mental rotation and structure-relationship mapping), which may partly explain the high ratings in visualization and engagement domains. However, cadaveric dissection remains essential for tactile experience, manual dexterity development, and professional attitudes that digital platforms cannot fully replicate, supporting a blended approach.

Although cadaveric dissection remains irreplaceable for its tactile feedback, appreciation of anatomical variation, and cultivation of professional attitudes toward human remains [16], the Pirogov table offers clear complementary benefits. Its strengths include safety, accessibility, and the capacity to demonstrate cross-sectional anatomy and pathological variants without ethical or logistical barriers. In resource-limited settings, where access to cadavers is restricted, the Pirogov table may serve as a practical adjunct to maintain educational quality and ensure equitable student access to anatomy training [17,18].

From an economic perspective, the integration of interactive anatomy tables warrants careful consideration of both initial investment and long-term operational costs. The Pirogov interactive anatomy table represents a substantial upfront capital expense (approximately USD 150,000–170,000 per unit); however, this investment includes lifetime hardware maintenance, battery replacement, and licensed software updates without additional subscription fees. Installation, user training, and technical onboarding are also provided without extra cost. During routine use, operational expenses are minimal and largely limited to electricity consumption and physical space allocation, without the need for dedicated technical staff.

In contrast, cadaver-based anatomy teaching entails continuous and cumulative expenditures related to infrastructure, preservation, and personnel. These include the procurement and operation of specialized refrigeration units, recurrent costs of chemical preservatives such as formaldehyde, disposal of used cadavers, and the employment of trained technical staff for specimen management and environmental safety. Over time, these recurring costs may equal or exceed the initial investment required for digital platforms, particularly in institutions with limited access to body donation programs.

This study has several limitations. First, knowledge was assessed using a 20-item multiple-choice test with acceptable psychometric performance, but it samples a limited breadth of anatomy content. The use of identical pre- and post-test items improves comparability but may introduce test–retest bias and inflate short-term scores due to item familiarity. Second, outcomes were measured immediately after instruction without delayed follow-up; therefore, long-term retention cannot be determined. Third, although randomization supported baseline comparability, we did not apply multivariable adjustment for potential confounders (e.g., baseline academic performance, gender, prior informal anatomy exposure). Cohort comparisons (Y1 vs Y2) were exploratory and involved unequal sample sizes, warranting cautious interpretation. Fourth, despite standardized delivery, residual instructor effects and cross-group contamination cannot be fully excluded. Fifth, we did not include performance-based outcomes (e.g., practical exams, OSCE-style stations, spatial ability testing), limiting conclusions to declarative knowledge. Finally, no formal cost or cost-effectiveness evaluation was conducted despite the high upfront and ongoing maintenance costs associated with digital platforms.

Despite these limitations, this is among the first controlled studies in Southeast Asia to rigorously evaluate the Pirogov interactive anatomy table. The findings support the integration of digital interactive platforms as valuable adjuncts to cadaveric dissection [19]. Future research should investigate long-term educational outcomes, clinical skill application, and cost-effectiveness to provide further guidance for curriculum development in anatomy education [20].

## Conclusion

Both cadaveric dissection and the Pirogov interactive anatomy table were associated with similar short-term improvements in anatomy knowledge in this cohort. Interactive tables may offer advantages in visualization and learner engagement, while cadaveric dissection remains essential for tactile learning and hands-on skill development. Future studies should evaluate long-term retention and include performance-based outcomes (e.g., practical examinations, OSCE-style anatomy stations, and spatial ability testing) to better determine the broader educational impact of integrating digital anatomy platforms into anatomy curricula.

## Supporting information

S1 File Pre-test and post-test instrument. A validated 20-item multiple-choice question (MCQ) test used to assess students’ anatomy knowledge immediately before (pre-test) and after (post-test) the instructional session. The instrument covers clinically relevant gross anatomy topics including upper limb, lower limb, neurovascular relationships, and key anatomical spaces/triangles. The same 20 items were administered in both assessments to ensure comparability across time points.
(DOCX)

S2 File Raw dataset for the study analyses. This file contains the anonymized raw dataset used for all statistical analyses in this study. Each row represents one participant and includes study variables collected at baseline and after the instructional session.
(XLSX)
