# Supplementary material for: Integration of the Pirogov interactive anatomy table into anatomy teaching: A comparative study with cadaveric dissection
Source: PLoS One. 2026 Jan 28;21(1):e0341597. doi: 10.1371/journal.pone.0341597 (PMC12851437; doi:10.1371/journal.pone.0341597)
Supplement: S1 File — A validated 20-item multiple-choice question (MCQ) test used to assess students’ anatomy knowledge immediately before (pre-test) and after (post-test) the instructional session. The instrument covers clinically relevant gross anatomy topics including upper limb, lower limb, neurovascular relationships, and key anatomical spaces/triangles. The same 20 items were administered in both assessments to ensure comparability across time points. (DOCX) [file pone.0341597.s001.docx]

Pre-test and Post-test

Question 1: The supraglenoid tubercle is the origin of:
A. Long head of the biceps brachii
B. Long head of the triceps brachii
C. Short head of the biceps brachii
D. Short head of the triceps brachii

Question 2: Which of the following statements about the femoral triangle is FALSE?
A. The medial border is the medial edge of the adductor longus
B. The lateral border is the medial edge of the sartorius
C. The base is the inguinal ligament
D. The apex is the point where the sartorius crosses the adductor longus

Question 3: The radius articulates with all of the following bones EXCEPT:
A. Humerus
B. Lunate
C. Scaphoid
D. Pisiform

Question 4: If a patient presents with deltoid muscle atrophy, which nerve is most likely injured?
A. Median nerve
B. Axillary nerve
C. Ulnar nerve
D. Radial nerve

Question 5: In the femoral triangle, the order of the femoral neurovascular bundle from medial to lateral is:
A. Femoral artery, femoral nerve, femoral vein
B. Femoral vein, femoral artery, femoral nerve
C. Femoral artery, femoral vein, femoral nerve
D. Femoral nerve, femoral artery, femoral vein

Question 6: In the anterior compartment of the leg, the anterior tibial artery usually runs between:
A. Tibialis anterior and extensor digitorum longus
B. Fibularis tertius and extensor digitorum longus
C. Extensor digitorum longus and extensor hallucis longus
D. Tibialis anterior and extensor hallucis longus

Question 7: Which of the following arteries is NOT a branch of the brachial artery?
A. Deep brachial artery
B. Nutrient artery
C. Circumflex humeral artery
D. Superior ulnar collateral artery

Question 8: Injury to the common fibular (peroneal) nerve results in:
A. Plantarflexion and inversion
B. Plantarflexion and eversion
C. Dorsiflexion and inversion
D. Dorsiflexion and eversion

Question 9: Cutaneous sensation of the anterior-medial thigh is supplied by which nerve?
A. Pudendal nerve
B. Obturator nerve
C. Lateral femoral cutaneous nerve
D. Ilioinguinal nerve

Question 10: Which branch of the axillary artery passes through the quadrangular space?
A. Anterior circumflex humeral artery
B. Posterior circumflex humeral artery
C. Lateral thoracic artery
D. Thoracoacromial artery

Question 11: In the popliteal fossa, which structure is the most superficial and lateral?
A. Common fibular (peroneal) nerve
B. Tibial nerve
C. Popliteal artery
D. Popliteal vein

Question 12: The tensor fasciae latae muscle is innervated by:
A. Superior gluteal nerve
B. Femoral nerve
C. Sciatic nerve
D. Inferior gluteal nerve

Question 13: Which of the following structures is located at the distal end of the tibia?
A. Tibial tuberosity
B. Soleal line
C. Fibular notch
D. Intercondylar eminence (posterior)

Question 14: Which structure passes through the adductor hiatus?
A. Femoral artery
B. Profunda femoris artery
C. Tibial nerve
D. Deep fibular (peroneal) nerve

Question 15: Which of the following arteries is a branch of the femoral artery?
A. Anterior tibial artery
B. Posterior tibial artery
C. Descending genicular artery
D. Middle genicular artery

Question 16: Above the pectoralis minor, the axillary artery gives rise to which branch?
A. Lateral thoracic artery
B. Thoracoacromial artery
C. Subscapular artery
D. Posterior circumflex humeral artery

Question 17: The posterior wall of the brachial canal is bounded by:
A. Skin and subcutaneous tissue
B. Humerus
C. Medial intermuscular septum
D. Lateral intermuscular septum

Question 18: In the anterior compartment of the forearm, the ulnar nerve innervates the flexor carpi ulnaris and:
A. Pronator teres
B. Pronator quadratus
C. Palmaris longus
D. Medial half of flexor digitorum profundus

Question 19: Injury to which of the following nerves may result in inability to extend the wrist?
A. Musculocutaneous nerve
B. Median nerve
C. Ulnar nerve
D. Radial nerve

Question 20: Which muscle accompanies the radial artery?
A. Palmaris longus
B. Brachioradialis
C. Extensor carpi radialis brevis
D. Extensor carpi radialis longus
